# Arsenic exposure, AS3MT polymorphism, and neuropsychological functioning among rural dwelling adults and elders: a cross-sectional study

**DOI:** 10.1186/1476-069X-13-15

**Published:** 2014-03-12

**Authors:** Melissa Edwards, James Hall, Gordon Gong, Sid E O’Bryant

**Affiliations:** 1 Department of Psychology, University of North Texas, 1155 Union Circle, Denton, TX 76203, USA; 2 Department of Psychiatry, University of North Texas Health Science Center, 3500 Camp Bowie Blvd, Fort Worth, TX 76107, USA; 3 Department of Rural and Community Health, Texas Tech Health Sciences Center, 3601 4th St, Lubbock, TX 79430, USA; 4 Department of Internal Medicine, University of North Texas Health Science Center, 3500 Camp Bowie Blvd, Fort Worth, TX 76107, USA; 5 Institute for Aging & Alzheimer’s Disease Research, University of North Texas Health Science Center, 3500 Camp Bowie Blvd, Fort Worth, TX 76107, USA

**Keywords:** Arsenic, AS3MT, Neuropsychology, Cognition

## Abstract

**Background:**

The aim was to examine the link between low-level arsenic exposure and cognitive functioning, and the potential role of a single nucleotide polymorphism (SNP A35991G, rs10748835) of the AS3MT gene in modifying this link.

**Methods:**

Data were analyzed on 526 participants from Project FRONTIER. Hierarchical linear regressions were created with neuropsychological raw index scores as the outcome variable and arsenic exposure and AS3MT SNP as different predictor variables.

**Results:**

Within the total sample, arsenic exposure was negatively associated with language (p < 0.001) and executive functioning (p < 0.001). Among those with the AA genotype of the AS3MT gene, arsenic levels were negatively associated with language (p < 0.001), attention (p = 0.01), and executive functioning (p = 0.04). Among those with the AG genotype, arsenic levels were positively associated with immediate (p = 0.04) and delayed memory (p < 0.001) and negatively associated with executive functioning (p = 0.03). Among those with the GG genotype, arsenic levels were negatively associated with visuospatial functioning (p = 0.02).

**Conclusions:**

Low-level arsenic exposure is associated with cognitive functioning; however, this association is modified by an AS3MT gene.

## Background

Arsenic, known as the Poison of Kings and the King of Poisons [1], is one of the most potent environmental toxicants [2]. Arsenic occurs in two different forms, inorganic and organic. Inorganic arsenic compounds are released from rocks or industrial and agricultural sources into the groundwater [3], which is the most common source of arsenic exposure. In 2006, the acceptable level of arsenic in drinking water changed in the United States from 50 parts per billion (ppb) to 10 ppb. This was in part due to a response to the increase of research studies showing that 50 ppb of arsenic in drinking water caused cancer in one out of every 100 people, which is more than 100 times that of any other drinking water contaminants at their respective maximum contaminant levels [4,5].

In addition to cancer, exposure to arsenic has also been linked to hypertension, arthrosclerosis, coronary heart disease, vascular diseases, stroke, metabolic syndrome [6-9], diabetes, cardiovascular disease, intellectual impairment in children, skin lesions, and other health related problems [6,10-13]. Though the standard level of arsenic in drinking water in the United States has decreased considerably, there is evidence to suggest that even exposure at low concentrations has negative health consequences.

Differences in arsenic metabolism are known to play a significant role in individual variability in arsenic-induced disease susceptibility [14]. Therefore, genetic variants in genes relevant to arsenic metabolism are considered to be responsible for the subsequent variation seen in arsenic metabolism. Specifically, a variant in arsenic (3+ oxidation state) methyltransferase (AS3MT), which is a key gene in the metabolism of arsenic, has been associated in several studies with increased arsenic methylation efficiency [14,15]. Findings have shown that arsenic-induced health effects may be especially deleterious in subsets of the population carrying susceptible variants of the genes relevant to arsenic metabolism [16]. Therefore, genetic differences in metabolic or biotransformation processes, even at a uniform level of cell or tissue exposure, may reflect differences in response rates because of variation in repair mechanisms, antioxidant status, and the availability of protective elements or other so far unrecognized factors [17]. The current study directly examined how arsenic exposure and the AS3MT gene polymorphism impact cognition.

Of the genetic factors that have been proposed to modulate arsenic associated effects among chronically exposed populations, the polymorphisms located within the AS3MT genes are among the most explored [15]. The differential prevalence of genetic polymorphisms responsible for arsenic metabolism have been previously described and it has been recommended that ethnicity is an important variable when considering the impact of arsenic exposure on health outcomes [18,19]. It has been proposed that variations in the AS3MT proteins, because of inherited genetic variants in the DNA sequence coding for amino acid, may influence the response to chronic arsenic exposure [15].

Many of the studies directly looking at the effect that arsenic has on cognitive functioning are conducted with samples of children, which found an inverse relationship between inorganic arsenic concentrations and attention, judgment, executive functioning, memory, processing speed, verbal abilities, and visual spatial abilities [13,20]. Among the few studies conducted with adults and elders, it was found that within a sample of rural dwelling adults, higher arsenic exposure (>10 μg/l estimated by GIS approach), was associated with lower global cognition when compared to those who had <10 μg/l arsenic exposure [21]. Another study examining a sample of rural dwelling adults and elders found a differential impact of current and long-term arsenic exposure [22]. In the latter study, it was found that there was a difference in cognitive functioning on the domains of language, visuospatial, and executive functioning for those who were exposed to current as opposed to long-term arsenic exposure [22]. Additionally, long-term low-level arsenic exposure was specifically found in the sample to impact cognitive functioning and result in lower performance on tasks of global cognition, visuospatial skills, language, executive functioning, processing speed, and immediate memory [22]. Overall, the focus of this study is to investigate how AS3MT gene polymorphisms impact the link between current arsenic exposure at low concentrations and cognitive functioning.

## Methods

### Participants

Participants consisted of community dwelling individuals from an ongoing rural healthcare study called Project FRONTIER (Facing Rural Obstacles Now through Intervention, Education & Research). This epidemiological study has been established with the following primary aims: (1) to evaluate the prevalence of cognitive impairment and dementia syndromes in a rural cohort, and (2) to examine healthcare disparities between ethnic groups residing within the county.

Individuals are eligible to participate in Project FRONTIER if (1) they are 40 years of age or older and (2) are a current resident of Cochran, Bailey or Parmer County, the three west Texas counties currently included in the study. All three neighboring counties reside on the Texas/New Mexico Border. Recruitment for this study was based on a community-based participatory research (CBPR) approach, which utilized a diversity of techniques that included visits with county leaders and contact with social groups as well as through the distribution of flyers, mail-outs, door-to-door solicitation, community presentations, and community recruiters. It has been previously shown that this CBPR approach yielded a sample comparable to that of the community at large [22].

All participants signed written informed consent prior to participation in the study. Once consent was obtained, participants undergo a medical examination by a clinician in their county of residence, a neuropsychological battery, an in-depth interview, and provide contact information for the completion of the informant telephone interview as outlined in the protocol for Project FRONTIER, which was approved by the Texas Tech University Health Sciences Center Institutional Review Board.

The mean age and years of education of the 527 participants was 61.50 (*sd* = 12.69; range = 40–96) and 10.86 (*sd* = 4.26; range = 0–20). Eighty percent (*n* = 424) of the sample was administered the neuropsychological examinations in English, the remainder was administered the assessment in Spanish. Fifty-seven percent of the sample reported their ethnicity to be non-Hispanic White whereas 42% (*n* = 223) reported to be Hispanic, with the majority (*n* = 207) of which identifying as Mexican American in origin. Of those participants genotyped for APOE (*n* = 527), 377 (71%) were APOE ϵ4 negative and 116 (22%) were APOE ϵ4 positive. Additionally, of the participants genotyped for AS3MT (*n* = 527), 81(15%) had the homozygous AA allele, 255 (48%) had the heterozygous AG allele, and 155 (29%) had the homozygous GG allele. GIS-estimated mean current groundwater arsenic level was 6.42 μg/l (*sd* = 2.99, range = 2.19-15.25). Descriptive statistics are presented in Table 1.

**Table 1:** Descriptive statistics

	**Mean ( *SD *)**	**Range**
Age	61.50 (12.69)	40 - 96
Years of education	10.86 (4.26)	0 - 20
Arsenic (μg/l)	6.42 (2.99)	2.19 - 15.25
Selenium (μg/l)	18.05 (10.62)	3.95 - 56.31
MMSE-World	27.61 (2.64)	12 - 30
EXIT25-Total	7.26 (4.61)	1 - 23
RBANS Immediate memory index	40.78 (9.20)	14 - 61
RBANS Visuospatial index	29.19 (6.08)	0 - 40
RBANS Language index	27.20 (5.16)	11 - 41
RBANS Attention index	46.92 (15.98)	9 - 100
RBANS Delayed memory index	35.50 (9.04)	10 - 60
RBANS Total index	86.34 (15.49)	49 - 136

### Neuropsychological measures

Participants enrolled in Project FRONTIER undergo a full neuropsychological battery as a part of the research study, which assesses for several cognitive domains and takes approximately two hours to complete. For purposes of addressing the link between arsenic exposure, AS3MT polymorphisms, and cognitive functioning, the results of the following tests in the battery were analyzed; 1) the Repeatable Battery for the Assessment of Neuropsychological Status (RBANS) [23] to assess for domains of attention, processing speed, verbal fluency, visuospatial abilities, immediate memory, and delayed memory, 2) the Executive Interview (EXIT25) was included to assess for executive functioning [24], the executive functioning tasks examined with the EXIT25 include verbal and design fluency, inhibition, mental fluency and a range of automatic behaviors [24] and 3) the Mini Mental Status Examination (MMSE) was included to assess for global cognition [25].

### Estimation of drinking water arsenic concentrations by geographic information system (gis) method

Current arsenic exposure will be determined through utilizing the geographic information system (GIS) method for calculating current residential groundwater arsenic levels. The ArcGIS software (release 9.2) developed by the Environmental Systems Research Institute [26] was utilized to estimate arsenic exposure by residential location for each participant. This method combines existing data on well-water arsenic levels from the Texas Water Development Board (TWDB) with current residential address. Arsenic levels from wells immediately surrounding the residential location are averaged and estimates of household arsenic level are generated based on distance from the wells using inverse-distance weighted (IDW) analysis.

### DNA

The Texas Tech University Health Sciences Center Department of Neurology laboratory conducted DNA assays. Genotyping of the AS3MT A35991G (rs10748835) polymorphism was performed by polymerase chain reaction (PCR) techniques. The PCR technique was performed in accordance with prior proposed techniques for analyzing the AS3MT polymorphism by Fujihara and colleagues [18]. The specific single nucleotide polymorphism (SNP) of AS3MT was selected for this study due to prior reports that the SNP is significantly associated with AS3MT methylation efficiency in human samples [18,19,27,28].

### Statistical analysis

The analyses were conducted through a series of hierarchical linear regressions utilizing SPSS 20.0. The raw scale scores for the RBANS indices and raw total scores for the EXIT25 and MMSE were utilized as outcome variables. The predictor variables for the analysis consisted of GIS-estimated current arsenic exposure and AS3MT polymorphism; covariates included variables previously shown to be significantly related to neuropsychological test scores (age, gender, years of education, language of test administration, APOEϵ4 carrier status), as well as factors previously shown to be related to arsenic exposure (estimated regional selenium levels). AS3MT was entered into the model as a categorical variable (GG, GA, or AA genotypes).

## Results

The distribution of scores was within normal limits and there were no violations of assumptions found within the hierarchical linear regressions. Correlations between the variables are presented in Table 2. Analysis of APOE and AS3MT for the study population revealed that they were not in Hardy Weinberg equilibrium.

**Table 2:** Correlations

	**1**	**2**	**3**	**4**	**5**
Male					
1. APOE ϵ4	-				
2. Age	0.07	-			
3. Years of education	0.04	-0.17*	-		
4. Selenium	-0.03	-0.13	-0.14	-	
5. Arsenic	-0.002	-0.08	-0.15	0.90**	-
*M*	0.23	63.43	10.37	18.63	6.51
*SD*	0.42	12.55	4.81	10.31	2.93
Female					
1. APOE ϵ4	-				
2. Age	-0.10*	-			
3. Years of education	-0.05	0.12*	-		
4. Selenium	-0.01	-0.08	-0.14**	-	
5. Arsenic	-0.01	-0.06	-0.17**	0.91**	-
*M*	0.23	60.60	10.94	17.28	6.25
*SD*	0.42	12.68	4.15	10.80	3.03

Note. *p < 0.05; **p < 0.01.

Current estimated groundwater higher arsenic exposure was found in the total sample to be significantly associated with poorer performance on tasks of language (RBANS Language Raw Index, *B* [*SE*] = -0.48 [0.15]; *p* < 0.001) and executive functioning (EXIT25, *B* [*SE*] = 0.49 [0.13]; *p* < 0.001) (see Table 3). Higher scores on tasks of executive functioning (EXIT25) indicate poorer level of cognitive functioning therefore a positive relationship indicates poorer performance scores as current GIS-estimated arsenic levels increase.

**Table 3:** Arsenic levels impact on cognitive functioning in the total sample

	***B***	***SE***	***t***	**p - value**
Total sample (*N* = 527)				
RBANS Immediate memory raw index	0.15	0.29	0.52	0.60
RBANS Visuospatial raw index	-0.39	0.19	-1.97	0.05
RBANS Language raw index	-0.48	0.15	-3.09	0.00*
RBANS Attention raw index	-0.66	0.39	-1.69	0.09
RBANS Delayed memory raw index	0.53	0.28	1.85	0.06
RBANS Total raw index	-0.82	0.96	-0.85	0.39
EXIT25	0.49	0.13	3.67	0.00*
MMSE- World	-0.14	0.08	-1.81	0.07

*Note.* Covariates included age, gender, education, language of administration (English or Spanish), selenium level and APOE ϵ4 presence (yes/no); *B* = unstandardized regression coefficient; *SE* = standard error.

*p < 0.05.

When the total sample was analyzed by AS3MT genotype (AA, AG, GG), higher current GIS-estimated groundwater arsenic exposure were found to differentially impact performance on tasks of cognitive functioning. Of those homozygous for the A allele of the AS3MT gene, current arsenic exposure levels were significantly associated with poorer performance on tasks of language (RBANS Language Index, *B* [*SE*] = -0.97 [0.28]; *p* < 0.001), attention (RBANS Attention Index, *B* [*SE*] = -1.90 [0.77]; *p* = 0.01), and executive functioning (EXIT25, *B* [*SE*] = -0.68 [0.33]; *p* = 0.04). Additionally, individuals with the GG genotype, current GIS-estimated arsenic level was significantly negatively associated with performance on visuospatial functioning (RBANS Visuospatial Index, *B* [*SE*] = -1.13 [0.48]; *p* = 0.02) only. However, of those with the AG genotype, higher current arsenic exposure level was significantly associated with better performance on tasks of immediate memory (RBANS Immediate Memory Index, *B* [*SE*] = 0.77 [0.38]; *p* = 0.04) and delayed memory (RBANS Delayed Memory Index, *B* [*SE*] = 1.07 [0.37]; *p* < 0.001) and with poorer performance on tasks of executive functioning (EXIT25, *B* [*SE*] = 0.40 [0.18]; *p* = 0.03) (see Table 4).

**Table 4:** Arsenic levels impact on cognitive functioning in the total sample split by AS3MT genotype

	**AA (*N*** **= 81)**				**AG (*N*** **= 255)**				**GG (*N*** **= 155)**			
	*B*	*SE*	*t*	p - value	*B*	*SE*	*t*	p - value	*B*	*SE*	*t*	p - value
RBANS Immediate memory	-0.84	0.70	-1.18	0.24	0.77	0.38	2.00	0.04*	-0.15	0.65	-0.23	0.81
RBANS Visuospatial	0.04	0.44	0.10	0.91	-0.34	0.24	-1.42	0.15	-1.13	0.48	-2.31	0.02*
RBANS Language	-0.97	0.28	-3.48	0.00*	-0.34	0.22	-1.51	0.13	-0.47	0.35	-1.34	0.18
RBANS Attention	-1.90	0.77	-2.46	0.01*	-0.05	0.52	-0.11	0.91	-0.88	0.94	-0.93	0.35
RBANS Delayed memory	0.32	0.60	0.53	0.59	1.07	0.37	2.84	0.00*	-0.51	0.68	-0.74	0.46
RBANS Total raw index	-3.34	1.99	-1.68	0.09	1.09	1.31	0.83	0.40	-2.76	2.26	-1.22	0.22
EXIT25	0.68	0.33	2.07	0.04*	0.40	0.18	2.18	0.03*	0.33	0.28	1.19	0.23
MMSE- World	-0.14	0.15	-0.89	0.37	-0.13	0.11	-1.24	0.21	-0.10	0.17	-0.56	0.57

*Note.* RBANS raw index scores are reported. Covariates included age, gender, education, language of administration (English or Spanish), selenium level and APOE ϵ4 presence (yes/no); *B* = unstandardized regression coefficient; *SE* = standard error.

*p < 0.05*.*

## Discussion

This study confirms prior work suggesting that higher low-level arsenic exposure reduces cognitive functioning, but extends previous work by demonstrating that this link varies by genetic inheritance. The results also demonstrated that low-level arsenic exposure significantly impacted cognition since the mean value of groundwater arsenic concentration estimated by the GIS approach was below the current allowable level of 10 ppb. These findings were consistent with the findings from Gong and colleagues [21], which demonstrated that low-levels of arsenic exposure are linked with poorer performance on tasks of cognitive functioning. However, Gong and colleagues [21] only analyzed the effects of arsenic exposure on global cognitive functioning, while this current study expanded the cognitive functioning domains to include language, visuospatial, and executive functioning. Gong and colleagues [21] also split their sample based on low-level exposure gradients, which this study did not do and based on the relatively limited variability within global cognitive functioning scores and may explain why this study did not demonstrate a significant association between low-level arsenic and global cognitive functioning. Moreover, this study yielded results similar to that of a study conducted by O’Bryant and colleagues [22], where higher levels of long-term low-levels of GIS-estimated arsenic were found to be associated with reduced language, visuospatial, and executive functioning; however, O’Bryant and colleagues did not study other aspects of cognitive functioning.

Additionally, this study demonstrated the significance that genetic inheritance and environmental factors can play related to differential susceptibility to neurotoxins. The results indicate a significant differential relationship between cognitive functioning and arsenic exposure, even with the mean arsenic exposure level found within the sample to be under the current acceptable level set by the Environmental Protection Agency (EPA). Also, the study utilized a population based in a rural locale in West Texas along the Texas-New Mexico border, which was selected based on its stepwise linear increase in arsenic exposure. The selected locations allowed the study to analyze arsenic exposure at increased levels within the same participant pool. The study was further able to provide insight into the differential genetic susceptibility within a population sample with less diverse genetic sampling.

Furthermore, the results also demonstrated a gene-environment interaction such that the impact of arsenic on cognitive functioning fluctuated by AS3MT SNPs (A35991G, rs10748835). Specifically, higher arsenic exposure was associated with lower language and attention abilities and lower executive functioning only among those with the AA genotype, and was associated with lower visuospatial functioning only among those with the GG genotype. Additionally, among those with the AG genotype, arsenic exposure was associated with decreased executive functioning; however, arsenic exposure was also found to be positively associated with immediate and delayed memory raw indices. The mechanisms are unknown.

## Conclusions

With the ever-growing aging population, deficits related to cognitive functioning will result in an increased problem posed by a significant proportion of the population at large. Therefore, risk factors associated with identifying inter-individual variation can help to earlier assess potential subsets of the population for which cognitive deficits may differentially impact. Cognitive functioning is related to a myriad of tasks conducted on a day-to-day basis including finances, medical decisions, and overall level of general functioning and therefore deficits impact not only the health of an individual but potentially others who may be dependent upon them. Often deficits of cognitive functioning transform into more severe forms of cognitive dysfunction such as dementia. Therefore, it is essential to help identify potential pre-cursers that could help to enable healthcare providers with the tools to provide early interventions in order to slow down the progression of the cognitive deficits.

One of the limitations of the study was that this study utilized a sample with an overall mean GIS-estimated arsenic exposure level below that of the current acceptable exposure level of 10 ppb set by the EPA. The mean arsenic exposure level of the sample was 6.42 ppb with the highest estimate found around 15 ppb. Though lower arsenic levels are a limitation of this study, it did allow for the exploration of health consequences related to arsenic exposure below what is currently in the acceptable range, thereby denoting adverse health implications related to the current EPA limit. Additional limitations of the study include the absence of information regarding water and food consumption patterns as well as source of water (groundwater, bottled, surface) among the sample. Water samples are being collected at this time for purposes of addressing this limitation.

Furthermore, there was a limited range in genetic variance due to the rural population utilized, such that the rural populations within the three West Texas counties used in the sample were less genetically diverse than what would be found within that of an urban population. The biggest limitation was that it is unfeasible to provide a comparison control group. Since everyone is essentially exposed to arsenic to some extent, there is not a population that is readily available to be evaluated without some degree of arsenic exposure or a population that can be readily examined with similar high levels of arsenic exposure within the locale selected for this study. Therefore, conditions are unable to be manipulated as would have been desired for a controlled study.

Future research should look at examining current GIS-estimated arsenic exposure, AS3MT polymorphism, and cognition within an urban sample to see if the findings converge. Additionally, future studies might serve to expand upon the level of arsenic exposure and include cross validating the results within a sample that is exposed to higher levels of arsenic (at or above EPA acceptable standards) thereby addressing a current limitation to this study of having low-level arsenic exposure. Examining the impact of group differences with high exposure versus low arsenic exposure will allow for further analyses to be conducted on the impact of arsenic on factors related to aging and explore if the current EPA limit for arsenic in groundwater should be lowered. Further research could also look at the arsenic exposure, AS3MT polymorphisms, and cognition link in a cognitively impaired sample to test the arsenic exposure hypothesis further. The current study underscores the need to investigate the biological and environmental factors that can impact the results of neuropsychological testing.

## Abbreviations

PPB: Parts per billion; AS3MT: Arsenic (3+ oxidation state) methyltransferase; CBPR: Community-based participatory research; FRONTIER: Facing rural obstacles now through intervention, education, & research; RBANS: Repeatable battery for the assessment of neuropsychological status; EXIT25: Executive interview 25 items; MMSE: Mini- mental status examination; GIS: Geographic information system; IDW: Inverse-distance weighted; PCR: Polymerase chain reaction; SNP: Single nucleotide polymorphism.

## Competing interests

The authors declare that they have no competing interests.

## Authors’ contributions

ME drafted the manuscript, conducted statistical analyses and contributed to the discussion. JH and GG provided statistical support, edited the manuscript and contributed to the discussion. SEO drafted the manuscript, supervised statistical analyses, and contributed to the discussion. All authors read and approved the final manuscript.
